# Cortical stimulation in pharmacoresistant focal epilepsies

**DOI:** 10.1186/s42234-020-00054-4

**Published:** 2020-09-25

**Authors:** Jens Ellrich

**Affiliations:** 1 Medical Faculty, University of Erlangen-Nuremberg, Erlangen, Germany; 2 Precisis AG, Heidelberg, Germany

**Keywords:** Adherence, Anticonvulsant, Chronic subthreshold cortical stimulation, Epicranial cortical stimulation, Long-term depression, Neuromodulation, Responsive neurostimulation, Seizure, Transcranial direct current stimulation, Transcranial magnetic stimulation

## Abstract

Pharmacoresistance and adverse drug events designate a considerable group of patients with focal epilepsies that require alternative treatments such as neurosurgical intervention and neurostimulation. Electrical or magnetic stimulations of cortical brain areas for the treatment of pharmacoresistant focal epilepsies emerged from preclinical studies and experience through intraoperative neurophysiological monitoring in patients. Direct neurostimulation of seizure onset zones in neocortical brain areas may specifically affect neuronal networks involved in epileptiform activity without remarkable adverse influence on physiological cortical processing in immediate vicinity. Noninvasive low-frequency transcranial magnetic stimulation and cathodal transcranial direct current stimulation are suggested to be anticonvulsant; however, potential effects are ephemeral and require effect maintenance by ongoing stimulation. Invasive responsive neurostimulation, chronic subthreshold cortical stimulation, and epicranial cortical stimulation cover a broad range of different emerging technologies with intracranial and epicranial approaches that still have limited market access partly due to ongoing clinical development. Despite significant differences, the present bioelectronic technologies share common mode of actions with acute seizure termination by high-frequency stimulation and long-term depression induced by low-frequency magnetic or electrical stimulation or transcranial direct current stimulation.

## Background

Epilepsies are characterized by unpredictable seizures and affect more than 70 million people worldwide (Beghi 2020; Ngugi et al. 2010). Focal seizures are the predominant seizure type in children and adults with a proportion of about 68% (Forsgren et al. 1996). Seizures are primarily treated by anticonvulsant drugs (ACD) that typically strengthen inhibition and attenuate excitation of synaptic processing via pre- and postsynaptic mechanisms (Loscher and Schmidt 2012). Nearly two dozen ACDs with different mechanisms of action have been introduced over the past three decades with the aim of providing a better efficacy or safety profile than previous drugs (Chen et al. 2020). Although these recent drugs have advantages in terms of drug-drug interactions and teratogenicity, still a third of epilepsy patients suffer from recurrent seizures (Janmohamed et al. 2020). This pharmacoresistance is defined as failure of adequate trials of two tolerated, appropriately chosen, and used ACD schedules to achieve sustained seizure freedom (Janmohamed et al. 2020; Kwan et al. 2010; Tang et al. 2017). This definition includes and emphasizes that drug treatments are tolerated and used, indicating the impact of adverse events and adherence. A recent meta-analysis assessed the efficacy and tolerability of ACD monotherapies in randomized, double-blinded, parallel group studies in adults with newly diagnosed focal epilepsies (Lattanzi et al. 2019). Whereas 58% of patients achieved seizure freedom, more than 73% complained about treatment-emergent adverse events (TEAE). ACD treatment was discontinued in 13% due to TEAEs (Baulac et al. 2012; Baulac et al. 2017; Brodie et al. 2007; Trinka et al. 2018). ACD treatment can have a significant impact on quality of life and concomitant TEAEs may be more important determinants of quality of life than seizures themselves (Hamer et al. 2015; Luoni et al. 2011). Lack of benefit, TEAEs, and complicated drug regimens are among the most important factors associated with medication non-adherence in epilepsies (Malek et al. 2017). More than 40% of epilepsy patients are non-adherent with an associated increased mortality risk. Thus, a third of epilepsy patients suffer from ongoing seizures and even more from TEAEs (Billakota et al. 2020).

Pharmacoresistance and TEAEs designate a considerable group of patients with focal epilepsies that requires alternative treatments such as neurosurgical intervention and neurostimulation. Whereas neurosurgical resection can only be applied to non-eloquent brain areas in a minority of patients (Engel Jr. 2018), neurostimulation technologies such as vagus nerve stimulation (VNS) and deep brain stimulation (DBS) affect the cranial vagus nerve or the anterior thalamus, respectively. Both areas are rather remote from the seizure onset zone (SOZ) in focal epilepsies and, therefore, these neurostimulations are accompanied by systemic and unspecific side effects, as well as adverse events (Gonzalez et al. 2019; Salanova et al. 2015). Most frequent adverse events caused by active VNS cover hoarseness, cough, paresthesia, pain, dyspnea, and headache with incidences of up to 62% (Gonzalez et al. 2019). The clinical importance of asystole, severe bradycardia, and sleep apnea is still under discussion. The most frequent device-related adverse events with active DBS include pain, paresthesia, discomfort, sensory disturbance, memory impairment, and dizziness (Salanova et al. 2015). Depression events were reported in 37%, memory impairment in 27%. Thus, VNS and DBS, have rather different adverse events that are potentially related to the sites of stimulation. With RNS, depression and memory impairment are reported in 25 and 12.5%, respectively (Nair et al. 2020). These prevalences with RNS tend to be lower than with DBS. However, RNS involves in 69% of all implantations DBS leads and is performed in only 31% of patients exclusively by cortical strips localized over the SOZ (FDA 2013). This comparison may indicate that pure neurostimulation of the SOZ may have less systemic and unspecific side effects than remote stimulation by VNS and DBS.

Direct neurostimulation of SOZ in neocortical brain areas may specifically affect neuronal networks involved in epileptiform activity without remarkable adverse influence on physiological cortical processing in immediate vicinity. Penfield and Jasper first applied focal electrical stimulation to humans in order to terminate spontaneous seizures detected by electrocorticography at the time of resective surgery (Penfield and Jasper 1954). Especially in the last decade various neurotechnologies emerged from preclinical and clinical pioneer work aiming at individualized cortical stimulation therapies in pharmacoresistant focal epilepsies (Table 1).

**Table 1:** Bioelectronic technologies for cortical stimulation in focal epilepsies

Technology	TMS Transcranial Magnetic Stimulation	tDCS transcranial Direct Current Stimulation	RNS Responsive NeuroStimulation	CSCS Chronic Subthreshold Cortical Stimulation	ECS Epicranial Cortical Stimulation
Invasiveness	Noninvasive	Noninvasive	Invasive, Intracranial	Invasive, Intracranial	Invasive, Extracranial
Placement of electrode/coil	Cutaneous	Cutaneous	Subdural	Subdural	Epicranial, Subgaleal
Stimulation frequency	0.5–1 Hz	Direct current	100–200 Hz	2 Hz	100 Hz, 8 Hz
Mode of actions	LTD	Hyperpolarization, LTD	Seizure termination	LTD	Seizure termination, LTD
Clinical trials	Lefaucheur et al. 2020; Mishra et al. 2020	Lefaucheur et al. 2017; VanHaerents et al. 2020; Yang et al. 2020	Morrell and Group RNSSiES 2011	Lundstrom et al. 2019	EASEE-II PIMIDES-I n.d.

### Noninvasive cortical stimulation

#### Transcranial magnetic stimulation (TMS)

Two recent meta-analyses focused on the clinical efficacy of TMS in pharmacoresistant epilepsies (Lefaucheur et al. 2020; Mishra et al. 2020).

Mishra et al. included seven randomized controlled trials (RCT) that compared repetitive TMS with sham or placebo controls (Mishra et al. 2020). Statistical analyses demonstrated a significant reduction of seizure frequency and interictal epileptiform discharges with active low-frequency TMS (0.5 to 1 Hz) as compared to control groups. After TMS treatment, anticonvulsant efficacy faded and was unverifiable within weeks suggesting that TMS exerts only a short-term effect.

Lefaucheur et al. identified only one additional sham-controlled trial in epilepsies that applied repetitive low-frequency TMS (0.5 Hz) in the period from 2014 to 2018 as compared to the meta-analysis done in 2014 by the same group (Lefaucheur et al. 2020). The primary level of recommendation C, i.e. possible anticonvulsant efficacy, for low-frequency repetitive TMS in epilepsies from 2014 did not change in 2018. However, confounding factors such as differences in paradigms of TMS interventions, types and clinical profiles of epilepsies, and the number of ACDs taken by patients should be considered.

All meta-analyses emphasized the largest clinical trial that involved 60 patients with pharmacoresistant focal epilepsies in order to verify the potential therapeutic value of low-frequency TMS (0.5 Hz) on a localized epileptic focus (Sun et al. 2012). The randomized, single-blind, controlled parallel group study allocated 60 patients to two groups with different TMS intensities applying 20% or 90% of resting motor threshold. Seizure frequency and interictal EEG epileptic discharges were compared between the baseline and follow-up periods of up to 8 weeks after TMS. Seizures and interictal epileptiform discharges significantly decreased following 2-weeks high intensity TMS treatment as compared with the baseline level. For the patients, who received low intensity TMS, seizures and spikes in the follow-up period did not show any differences when compared with the baseline data. These results resemble data from a previous RCT in which TMS was applied to the individual epileptic focus in 21 patients, as well (Fregni et al. 2006).

Overall, the most recent meta-analysis suggested a significant beneficial effect of TMS on pharmacoresistant epilepsies reducing both the seizure frequency and interictal epileptiform discharges. However, potential anticonvulsant effects seem to be ephemeral and may require effect maintenance by ongoing repetitive TMS.

#### Transcranial direct current stimulation (tDCS)

A meta-analysis from 2017 identified 65 papers, including only 10 original clinical studies with 147 patients (Lefaucheur et al. 2017). Besides some case reports, 5 sham-controlled studies with crossover or parallel-arm design including 12 to 37 patients were addressed. No recommendation for cathodal tDCS of the epileptic focus or anodal tDCS of the left dorsolateral prefrontal cortex was given. A recent literature review concluded that study results of cathodal tDCS for refractory epilepsy were promising and suggested that cathodal tDCS may potentially decrease seizures in pharmacoresistant epilepsy patients (VanHaerents et al. 2020).

A very recent RCT aimed at the anticonvulsant effect of tDCS especially in patients with pharmacoresistant focal epilepsies (Yang et al. 2020). The multicenter clinical trial applied different tDCS paradigms to three groups of patients. Patients in group 2 received 20 min tDCS per day. Patients in the sham group (group 1) received no real stimulation but went through the same stimulation procedure as those in group 2, being attached with two electrodes for 20 min. For group 3, patients received a total of 40 min stimulation per day, which was equally separated by a 20 min interval. The primary outcome measurement was seizure frequency. The study consisted of 28 days baseline, 14 days treatment, and 56 days follow-up. The cathode was placed over the epileptogenic focus, and the current intensity was 2 mA. Seventy patients were included for the final analysis. There was a significant reduction in seizure frequency for both active tDCS groups compared with the sham group. Patients in group 2 showed a significantly greater reduction as compared to the sham group that lasted for 4 weeks. Patients in group 3 showed a significantly greater reduction as compared with the sham group that lasted for 5 weeks. Seizure frequency reduction in group 3 was significantly larger than in group 2. The authors concluded, that tDCS on 14 consecutive days significantly decreased seizure frequency in patients with pharmacoresistant focal epilepsies, with two daily 20 min stimulations being superior to a 20 min daily stimulation protocol (Yang et al. 2020).

In contrast to typical tDCS where sustained direct current stimulation is administered for at least 20 min, a slow-pulsed transcranial electrical stimulation protocol applied short cathodal direct current pulses of 100 ms duration with a frequency of 0.5 Hz to patients with pharmacoresistant focal epilepsies (Holmes et al. 2019). In all 7 subjects accurate targeting of the cortical focus of epileptic spikes was achieved by the combination of a high-resolution head conductivity model and a 256-channel dense EEG system. Interictal spikes were localized, and transcranial electrical stimulation targeted the cortical source of each subject’s principal spike population. Baseline EEG recording was followed by three 17 min trains of 500 cathodal pulses each, with trains separated by a 10 min rest interval. Continuous EEG recording for 3 h followed each of the five daily stimulation sessions and allowed for the assessment of the posttreatment spike rate. Targeted spikes were suppressed in five subjects, and nontargeted spikes were suppressed in four subjects. Epileptiform activity did not worsen.

### Invasive cortical stimulation

#### Responsive neurostimulation (RNS)

The implantable components of the RNS device include a cranially seated internal pulse generator connected to depth and/or cortical strip leads which are surgically placed at the seizure foci (Skarpaas et al. 2019). The neurostimulator continuously senses and monitors electrocorticographic activity at the seizure focus and provides responsive electrical stimulation when abnormal patterns are detected. Stimulation consists of current-controlled, charge-balanced biphasic pulses with the stimulation frequency being between 1 and 333 Hz, current between 0.5 and 12 mA, pulse width from 40 to 1000 μs, and a burst duration from 10 to 5000 ms. The most common stimulation settings in the clinical trials were 100–200 Hz stimulation frequency, 1.5–3 mA current, 160 μs pulse width, and 100–200 ms burst duration (Heck et al. 2014; Skarpaas et al. 2019). Usually, patients had 600–2000 detections per day. At typical burst durations, this adds up to about 6 min of stimulation per day.

A multi-center RCT demonstrated the safety and effectiveness of RNS. Over 3 months of the blinded period, the overall reduction in disabling seizure frequency in the treated patients (38%) was significantly larger than in the sham patients (17%) (Morrell and Group RNSSiES 2011). Seizure frequency reductions begin with initiation of treatment and continue over time, reaching median reductions of 75% after 9 years of treatment. Treatment with responsive cortical stimulation is also associated with an improvement in quality of life and cognitive function related to the functional area being treated.

#### Chronic subthreshold cortical stimulation (CSCS)

CSCS is an open-loop subthreshold stimulation via subdural electrodes that targets the SOZ (Kerezoudis et al. 2018; Lundstrom et al. 2019; Starnes et al. 2019). Potential candidates are identified while undergoing intracranial EEG monitoring. Seizure frequency as well as the frequency of interictal epileptiform discharges are used to assess the potential efficacy of multiple stimulation paradigms. First clinical data indicate positive effects on focal seizure frequency and severity (Kerezoudis et al. 2018; Starnes et al. 2019).

A recent retrospective analysis addressed potential interictal EEG biomarkers recorded during trial stimulation that may predict anticonvulsant efficacy of permanent CSCS (Lundstrom et al. 2019). When intracranial EEG monitoring advised against potential surgical resection of the SOZ, patients were offered a 1 to 4 days therapeutic trial of continuous electrical stimulation (biphasic; frequency 2–100 Hz; pulse width 90–450 μs; amplitude 1–6 V) targeting the SOZ and surrounding tissue using the already implanted temporary electrodes applied for invasive monitoring. The primary purpose of the trial stimulation was to optimize stimulation location and stimulation parameters via individual assessment of EEG epileptiform activity in response to stimulation. In the vast majority of 21 patients a stimulation frequency of about 2 Hz during invasive monitoring effectively reduced interictal epileptiform discharges and seizure activity. Permanent stimulation hardware was implanted when intracranial EEG electrodes were explanted. Three months after stimulation initiation, the responder rate with at least 50% seizure reduction was 79%, the median reduction in seizure frequency was 93%. With a median follow-up of 27 months, in the most recent 3 months period the responder rate was 89% with a median reduction in seizure frequency of 100%. About 40% of patients were free of disabling seizures for a 12 months period or longer. It was demonstrated that stimulation induced decreases in delta (1–4 Hz) power and increases in alpha and beta (8–20 Hz) power during trial stimulation correlated with improved long-term clinical outcomes. CSCS has been implemented via off-label usage of FDA-approved hardware. The authors suggested that CSCS may be an effective alternative approach in the treatment of pharmacoresistant focal epilepsies and that short-term stimulation-related changes in EEG spectral power may be a useful interictal biomarker that seems to relate to long-term clinical outcome (Lundstrom et al. 2019).

#### Epicranial cortical stimulation (ECS)

**Fig. 1:**
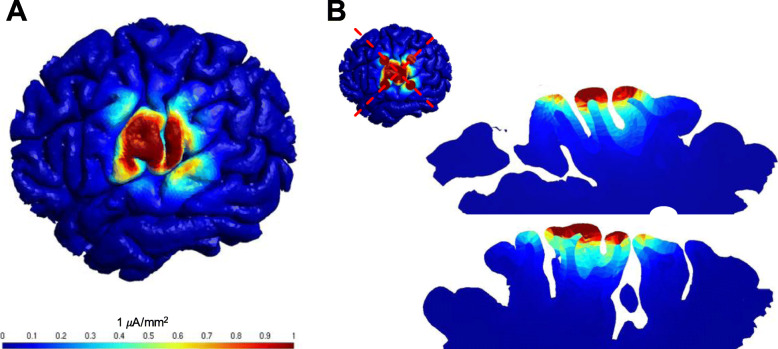
Electrical field simulation via finite element method based on a high-resolution head model. Electrical stimulation of the left parieto-temporal cortex is applied by an epicranial array consisting of five electrodes arranged in a pseudo-Laplacian geometry, with a central electrode surrounded by four peripheral electrodes. **a** Top view of the current density distribution. **b** Cross sections in two perpendicular layers with intracortical current density distribution

Epicranial application of electrical stimulation may combine minimal invasiveness with sustained efficacy of neuromodulation in focal epilepsies. Preclinical experiments in rodents applied epicranially implanted electrodes in order to selectively stimulate the motor cortex. Experiments demonstrated focused limb movements by ECS with a design of concentric ring electrodes according to Laplace (Khatoun et al. 2019). The combination of a minimally invasive epicranial electrode and an internal pulse generator recently made the first move into clinical development (Schulze-Bonhage 2019). The epicranial array consists of five electrodes arranged in a pseudo-Laplacian geometry, with a central electrode surrounded by four peripheral electrodes allowing precise individual targeting of defined neocortical brain areas (Alam et al. 2016; Saturnino et al. 2015). Electrical field simulation via finite element method based on a high-resolution head model indicates substantial field strength as compared to noninvasive neurostimulation (Fig. 1) (Saturnino et al. 2015). In contrast to other invasive devices, the electrode is not placed intracranially but in the subgaleal space between the scalp and skull. Based upon clinical outcomes of noninvasive cathodal tDCS and intracranial RNS in focal epilepsies, ECS combines two stimulation paradigms in ongoing clinical trials (EASEE-II n.d.; PIMIDES-I n.d). Asymmetric, rectangular pulses with durations of 20 ms and 100 ms of cathodal and anodal half-waves, respectively, are applied with a frequency of 8 Hz for 20 min a day resembling tDCS. AC bursts of 500 ms duration consist of biphasic, rectangular pulses (320 μs, 100 Hz) and are applied every 2 minutes throughout the day; Daily duration of AC bursts adds up to 6 min which is similar to RNS. Both stimulation paradigms are individually adjusted to subthreshold stimulus intensities below 4 mA. Besides the open-loop subthreshold stimulation that is delivered at regular intervals throughout the day in the evaluation period of the clinical trials, the PIMIDES-I study offers additional bolus stimulation in order to provide patients the ability to manually deliver an additional dose of therapy in an effort to stop or shorten a seizure once it starts (PIMIDES-I n.d.). Both clinical trials include more than 30 patients suffering from pharmacoresistant focal epilepsies and are ongoing, first results are expected in 2021.

### Mode of action

#### Seizure termination

Direct electrical stimulation has been applied to the cortex for mapping purposes for about 90 years (Luders et al. 1988). Cortical localization stimuli may provoke epileptiform afterdischarges, and these can evolve into clinical seizure. These afterdischarges are a preliminary stage of seizures and, therefore, used as a model of epileptiform activity. High-frequency cortical stimulation of 50 Hz was performed for clinical localization purposes using subdural electrodes implanted in patients undergoing preresection evaluations for treatment of pharmacoresistant seizures (Lesser et al. 1999). When high-frequency stimulation produced afterdischarges, brief bursts of additional stimulation were applied in order to prove its effectiveness in aborting afterdischarges in 17 epilepsy patients. Afterdischarges significantly decreased in duration after the application of brief bursts. These results support the idea that high-frequency electrical stimulation, applied in an appropriate manner at seizure onset, could abort seizures in humans. Further studies in epilepsy patients have also reported on shortening or termination of epileptiform discharges by high-frequency electrical cortical stimulation (Chkhenkeli et al. 2004; Motamedi et al. 2002). RNS mainly is based upon the termination of epileptiform brain activity by high-frequency cortical stimulation (Morrell 2006).

Obviously, high-frequency electrical stimulation is able to provoke opponent effects in the brain, it might induce epileptiform activity but may also terminate those discharges and subsequently initiating seizures as well (Lesser et al. 1999). Mechanisms of synaptic plasticity delineated by the model of long-term potentiation (LTP) are similar to those underlying epileptogenesis by kindling (Meador 2007; Weiss et al. 1995). Both kindling and LTP are most effectively induced by high-frequency stimulation and share overlapping molecular mechanisms.

#### Long-term depression (LTD)

Kindling is the progressive development of seizures to a previously subconvulsant stimulus (Goddard et al. 1969). Rats were kindled by daily application of high-frequency electrical stimulation to the amygdala (Weiss et al. 1995). Kindling was characterized by decreased thresholds and increased durations of afterdischarges and seizures. Additional low-frequency stimulation (LFS; 1 Hz, 15 min) blocked the development and progression of afterdischarges and seizures in seven out of eight animals. In fully kindled animals once daily LFS for 1 week suppressed seizures when the kindling stimulation was resumed. It is suggested that LFS effectuates long-lasting anticonvulsant effects mediated by LTD (Weiss et al. 1995).

Although LTD has been investigated intensively in animal models (Braunewell and Manahan-Vaughan 2001; Ellrich 2004; Kemp and Bashir 2001), only few studies have focused on possible mechanisms involved in this synaptic plasticity model in humans. Electrical LFS induced LTD of brainstem reflexes (Ellrich and Schorr 2002; Schorr and Ellrich 2002) and somatosensory brain processing assessed by electrophysiological (Ellrich and Schorr 2004; Jung et al. 2009; Rottmann et al. 2008), psychophysical (Rottmann et al. 2010a), and imaging methods (Rottmann et al. 2010b) in humans.

The common denominator in the vast majority of LTD studies is the application of LFS in the range of 0.5 to 3 Hz. This applies to noninvasive TMS with main frequencies of repetitive stimulation between 0.5 and 1 Hz (Lefaucheur et al. 2020; Mishra et al. 2020) and intracranial CSCS with 2 Hz LFS (Lundstrom et al. 2019) in patients with focal epilepsies.

LFS induces LTD by N-Methyl-D-aspartate (NMDA) receptor activation and the subsequent slight increase in Ca^2+^ activating calcineurin which stimulates protein phosphatase 1 (PP1) (Xia and Storm 2005). Activation of PP1 catalyzes the dephosphorylation of several proteins.

Noninvasive cathodal tDCS is suggested to provoke transient and long-term effects on cortical excitability (Antal et al. 2017; Luu et al. 2016; Nitsche et al. 2003; Nitsche and Paulus 2000; Stagg et al. 2018) and epileptiform discharges (Holmes et al. 2019; VanHaerents et al. 2020; Yang et al. 2020). Whereas acute effects of cathodal tDCS are due to transient hyperpolarization of cortical neurons (Nitsche et al. 2003; Nitsche and Paulus 2000), human experimental studies provide evidence that long-term effects base upon LTD (Stagg et al. 2018). Pharmacological studies show that a blockade of NMDA receptors prevents sustained tDCS-induced inhibition in human and experimental studies (Chang et al. 2015; Nitsche et al. 2003). Blockade of PP1 by okadaic acid in the same experimental model suspended long-term inhibition of cathodal tDCS and spared acute effects (Chang et al. 2015). Low-frequency magnetic or electrical stimulation and cathodal tDCS seem to share LTD as a common mode of action. Depending on experimental model and stimulation parameters, LTD effects may last hours to weeks. Sustainability of LTD may define noninvasive and invasive stimulation paradigms.

## Conclusions

Electrical or magnetic stimulations of cortical brain areas for the treatment of pharmacoresistant focal epilepsies emerged from preclinical studies and experience from intraoperative neurophysiological monitoring in patients (Table 1). Noninvasive low-frequency TMS and cathodal tDCS are suggested to be anticonvulsant, however, potential effects are ephemeral and may require effect maintenance by ongoing stimulation. Invasive RNS, CSCS, and ECS cover a broad range of different emerging technologies with intracranial and epicranial approaches that still have limited market access partly due to ongoing clinical development. Despite significant differences, the present bioelectronic technologies share common mode of actions with acute seizure termination by high-frequency stimulation and LTD induced by low-frequency magnetic or electrical stimulation or tDCS. Focused cortical stimulation with synergistic mode of actions seems to be a promising option for sustained anticonvulsant treatment of one third of epilepsy patients that suffer from ongoing seizures and adverse effects of drug treatment.

## Data Availability

Not applicable.

## Abbreviations

ACD: Anticonvulsant Drug
CSCS: Chronic Subthreshold Cortical Stimulation
DBS: Deep Brain Stimulation
ECS: Epicranial Cortical Stimulation
EEG: ElectroEncephaloGraphy
LFS: Low-Frequency Stimulation
LTD: Long-Term Depression
LTP: Long-Term Potentiation
NMDA: N-Methyl-D-Aspartate
PP1: Protein Phosphatase 1
RCT: Randomized Controlled Trial
RNS: Responsive NeuroStimulation
SOZ: Seizure Onset Zone
tDCS: transcranial Direct Current Stimulation
TEAE: Treatment-Emergent Adverse Event
TMS: Transcranial Magnetic Stimulation
VNS: Vagus Nerve Stimulation
